# Endoscopic screening of the upper gastrointestinal tract for second primary tumors in patients with head and neck cancer in a Western country

**DOI:** 10.1055/a-2111-5935

**Published:** 2023-07-25

**Authors:** Laurelle van Tilburg, Steffi E. M. van de Ven, Pieter Jan F. de Jonge, Wilmar de Graaf, Manon C. W. Spaander, Suzan Nikkessen, Jose A. Hardillo, Aniel Sewnaik, Dominiek A. Monserez, Hetty Mast, Stijn Keereweer, Marco J. Bruno, Robert J. Baatenburg de Jong, Arjun D. Koch

**Affiliations:** 1 Department of Gastroenterology and Hepatology, Erasmus MC Cancer Institute, University Medical Center, Rotterdam, the Netherlands; 2 Department of Otorhinolaryngology and Head and Neck Surgery, Erasmus MC Cancer Institute, University Medical Center, Rotterdam, the Netherlands; 3 Department of Oral and Maxillofacial Surgery, Erasmus MC Cancer Institute, University Medical Center, Rotterdam, the Netherlands

## Abstract

**Background** Patients with head and neck squamous cell carcinoma (HNSCC) can develop second primary tumors (SPTs) in the esophagus. Endoscopic screening could lead to detection of SPTs at early stages and improve survival.

**Methods** We performed a prospective endoscopic screening study in patients with curably treated HNSCC diagnosed between January 2017–July 2021 in a Western country. Screening was performed synchronously (< 6 months) or metachronously (≥ 6 months) after HNSCC diagnosis. Routine imaging for HNSCC consisted of flexible transnasal endoscopy with positron emission tomography/computed tomography or magnetic resonance imaging, depending on primary HNSCC location. The primary outcome was prevalence of SPTs, defined as presence of esophageal high grade dysplasia or squamous cell carcinoma.

**Results** 202 patients (mean age 65 years, 80.7 % male) underwent 250 screening endoscopies. HNSCC was located in the oropharynx (31.9 %), hypopharynx (26.9 %), larynx (22.2 %), and oral cavity (18.5 %). Endoscopic screening was performed within 6 months (34.0 %), 6 months to 1 year (8.0 %), 1–2 years (33.6 %), and 2–5 years (24.4 %) after HNSCC diagnosis. We detected 11 SPTs in 10 patients (5.0 %, 95 %CI 2.4 %–8.9 %) during synchronous (6/85) and metachronous (5/165) screening. Most patients had early stage SPTs (90 %) and were treated with curative intent with endoscopic resection (80 %). No SPTs in screened patients were detected with routine imaging for HNSCC before endoscopic screening.

**Conclusion** In 5 % of patients with HNSCC, an SPT was detected with endoscopic screening. Endoscopic screening should be considered in selected HNSCC patients to detect early stage SPTs, based on highest SPT risk and life expectancy according to HNSCC and comorbidities.

## Graphical abstract

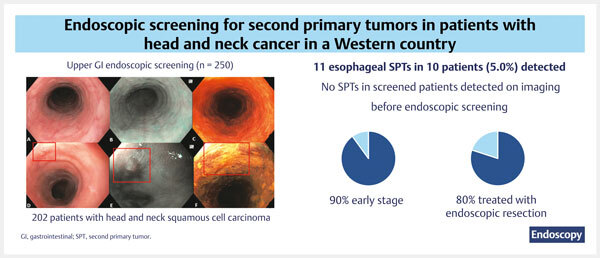

## Introduction

In Western countries, approximately 11 % of patients with head and neck squamous cell carcinoma (HNSCC) develop a second primary tumor (SPT) 1 . These SPTs are often located in the upper aerodigestive tract, which consists of the head and neck region, lungs, and esophagus 1 . In particular, esophageal SPTs frequently remain undetected until reaching advanced stages and are therefore associated with decreased survival rates 2 .

Endoscopic screening of the upper gastrointestinal (GI) tract allows for timely detection of SPTs at early and curable stages 3 4 . Early stage SPTs can be treated with minimally invasive endoscopic resection, potentially improving the survival of patients with HNSCC 5 . Consequently, endoscopic screening in patients with HNSCC is routinely implemented in countries with a high incidence of esophageal and gastric cancer, such as China and Japan 6 7 8 . In Asian countries, several screening studies in patients with HNSCC have been conducted, reporting a prevalence of 3 %–41 % esophageal SPTs 7 9 10 11 12 .

Conversely, in Western countries, the incidence of esophageal squamous cell carcinoma is relatively low (age-standardized incidence rate of < 3.5 per 100 000) compared with Asia 8 . Thus, the results from Asian studies in patients with HNSCC should not be generalized, and so far, most Western countries have not implemented routine screening for SPTs in the upper GI tract 13 14 . Data from screening studies originating from Western countries are scarce and consist mainly of studies with small numbers of patients with HNSCC. These published studies report the detection of esophageal SPT in up to 10 % of patients with HNSCC 3 15 16 17 . Risk factors for the development of SPTs in patients with HNSCC include human papillomavirus-negative tumors located at the oropharynx or hypopharynx, and patients with excessive alcohol consumption and tobacco use 2 18 .

The detection of SPTs can be divided into synchronous (within 6 months) and metachronous (after more than 6 months), according to the time interval between HNSCC diagnosis and endoscopic screening. In 2019, our group started a prospective screening program for synchronous SPTs in the upper GI tract in patients with HNSCC 3 . The current study is an extension of the aforementioned study, presenting the results of both synchronous and metachronous endoscopic screening in a selected group of patients with HNSCC in a Western country.

## Methods

### Study design and patients

We performed a prospective endoscopic screening study of patients who were diagnosed with HNSCC between January 2017 and July 2021 in a tertiary referral center in the Netherlands. Patients with HNSCC with an increased risk of SPTs, based on previously published studies 4 , were eligible for endoscopic screening. This consisted of patients with HNSCC located in the oropharynx, hypopharynx, and other subsites, combined with alcohol abuse (≥ 14 units per week for males and ≥ 7 units per week for females) 3 19 . The eligibility criteria and results of patients included in the synchronous screening program have been described in detail previously 3 . Exclusion criteria were 1) cancer at an incurable stage, 2) upper GI cancer detected before endoscopic screening, 3) severe comorbidities, preventing patients from undergoing endoscopic screening, and 4) follow-up performed in other hospitals. Patients with human papillomavirus-associated oropharyngeal carcinoma were also excluded, as these patients often present without common risk factors for SPTs, such as smoking and alcohol use, and are known to have a lower risk profile for SPTs 20 . High risk human papillomavirus testing was performed in patients with oropharyngeal carcinoma with immunohistochemistry for a surrogate p16 marker 21 .

### HNSCC staging and follow-up

All included patients received routine staging and follow-up for HNSCC, according to current Dutch guidelines 21 . In the Netherlands, care for all patients with HNSCC is centralized in 14 expert centers, which perform the diagnostic work-up and discuss treatment options in multidisciplinary meetings. The diagnostic work-up of HNSCC includes a panendoscopy (i. e. flexible transnasal endoscopy examining the oral cavity, nasopharynx, hypopharynx, oropharynx, and larynx) and computed tomography (CT) scan or magnetic resonance imaging (MRI) scan, depending on HNSCC location. Patients with an increased risk of distant metastasis (i. e. patients with low jugular, bilateral or N3 lymph node metastasis) receive a positron emission tomography/CT (PET/CT) scan. Routine follow-up visits after HNSCC treatment include physical examination and pandendoscopy. The aim of follow-up for HNSCC is early detection of disease recurrence and SPTs in the head and neck region. In cases of suspected HNSCC recurrence or SPTs, staging and treatment is performed within daily clinical practice.

### Endoscopic screening

Endoscopic screening was performed with high definition endoscopes by expert endoscopists (A.D.K., M.C.W.S., P.J.F.dJ, S.N., and W.dG.), who each had more than 5 years’ experience in the detection of neoplasia in the upper GI tract. All endoscopists participated in a dedicated upper GI screening program and had extensive experience in the detection of premalignant lesions in the upper GI tract. Endoscopic screening was performed with high definition white-light endoscopy (WLE), optical chromoendoscopy (narrow-band imaging [NBI]), and Lugol’s staining. First, the mucosae of the stomach, duodenum, and esophagus were carefully inspected with WLE. Second, the esophageal and gastric mucosae were inspected again with NBI. After switching back to WLE, 10–30 mL of Lugol’s staining (1.2 % iodine solution) was applied to the esophageal mucosa with a spray catheter or syringe. Visible lesions were classified according to the Paris and intrapapillary capillary loop classifications, and assessed for endoscopic resectability 22 . No routine target biopsies were taken of SPTs amenable to endoscopic resection and no random biopsies of the esophagus were taken. In cases of suspected SPT that could not be treated with endoscopic resection, targeted biopsies were taken. Adverse events that occurred as a result of endoscopic screening were recorded.

### Timing of endoscopic screening

All included patients received at least one screening endoscopy. The study cohort consisted of three screening groups: synchronous screening only, synchronous with subsequent metachronous screening, and metachronous screening only. First, synchronous screening was performed in included patients diagnosed with HNSCC between February 2019 and February 2020 3 . Second, among patients that had at least 1 year of follow-up for HNSCC and fulfilled the eligibility criteria, we performed a follow-up screening endoscopy (i. e. metachronous screening 1 year after synchronous screening). Third, it was decided to include eligible patients diagnosed between January 2017 and February 2019, and between February 2020 and July 2021 to increase patient inclusion in the metachronous screening cohort. These patients were approached for metachronous screening 1–5 years after HNSCC diagnosis (i. e. metachronous screening alone).

### Second primary tumors

SPTs were defined as the presence of esophageal high grade dysplasia (HGD) or esophageal squamous cell carcinoma. The detection of squamous low grade dysplasia (LGD), a precursor lesion of esophageal squamous cell carcinoma, was also monitored. All cases of LGD were reviewed by an expert team of three experienced upper GI pathologists until consensus regarding the grade of dysplasia was reached. Lesions larger than 5 mm detected during endoscopic screening with WLE, NBI, and/or Lugol’s staining, were considered suspicious for SPT or LGD. In cases of confirmed SPT, treatment was discussed in a multidisciplinary tumor board meeting with the gastroenterologist, GI surgeon, head and neck surgeon, radiologist, and oncologist. Treatment options for SPTs included endoscopic mucosal resection (EMR) or endoscopic submucosal dissection (ESD), surgery, and chemoradiotherapy. Other findings, including GI tract cancers such as esophageal adenocarcinoma and gastric cancer, Barrett’s esophagus, reflux esophagitis (according to the Los Angeles classification), and gastritis, were treated as per standard clinical care.

### Study end points

The primary end point was the prevalence of SPTs detected during endoscopic screening of the upper GI tract. Secondary end points were 1) histology and tumor stage of SPTs, 2) time to detection, treatment, and outcomes of patients with HNSCC and SPTs, and 3) proportion of SPTs detected during a follow-up endoscopy after 1 year. Additionally, we also report on the proportion, histology, and stage of SPTs diagnosed on imaging for HNSCC or in symptomatic patients.

### Statistics and ethics

Descriptive statistics are presented as mean with SD, median with interquartile range (IQR), and count with percentage, according to the nature of the data. The detection rates of SPTs were reported with 95 %CIs, and follow-up data were obtained to December 2022. Statistical analysis was performed using IBM SPSS for Windows version 28 (IBM Corp., Armonk, New York, USA). Informed consent was obtained from all included patients. The study protocol conformed to the ethical guidelines of the 1975 Declaration of Helsinki. The study was registered in the Netherlands Trial Register (NL7299) and approved by the Medical Ethical Review Committee of the Erasmus Medical Center, Rotterdam, the Netherlands (MEC-2018–1243).

## Results

### Patients

**Fig. 1:**
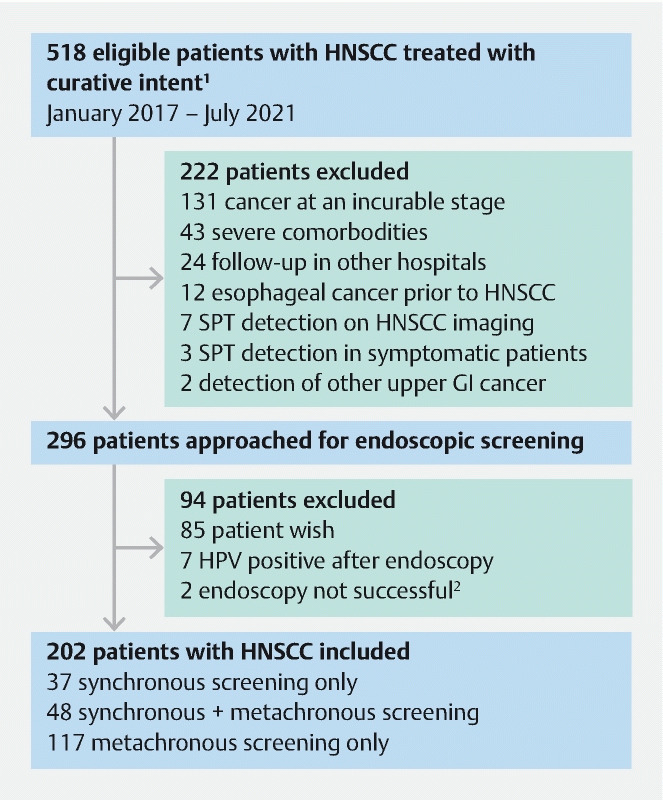
Flowchart of patient inclusion. ^1^ Patients diagnosed with head and neck squamous cell carcinoma between February 2019 and February 2020 were approached for synchronous and metachronous screening, if fulfilling the eligibility criteria. ^2^ Endoscopy not successful, due to neopharyngeal stricture (n = 1) and need for sedation (n = 1). HNSCC, head and neck squamous cell carcinoma; GI, gastrointestinal; HPV, human papillomavirus; SPT, second primary tumor.

A total of 518 eligible patients were diagnosed with HNSCC between January 2017 and July 2021 ( Fig. 1 ; see also **Fig. 1 s** in the online-only Supplementary material). Of these patients, 222 patients were excluded because of cancer at an incurable stage (n = 133), severe comorbidities (n = 43), treatment and follow-up in other hospitals (n = 24), a history of esophageal cancer before HNSCC diagnosis (n = 12), and detection of an SPT before endoscopic screening could be performed (n = 10). In total, 296 patients with HNSCC were approached for inclusion, of whom 202 (68.2 %) were included and underwent successful endoscopic screening. Most patients included were male (80.7 %) and the median patient age was 65 years (IQR 59–69 years) ( Table 1 , **Table 1 s** ). The majority of the patients consumed alcohol (78.2 %) and were current (43.6 %) or former (51.0 %) tobacco smokers. The HNSCC of included patients was located in the oropharynx (31.9 %), hypopharynx (26.9 %), larynx (22.2 %), and oral cavity (18.5 %).

**Table 1:** Baseline and head and neck characteristics in the included patients.

Patient characteristics	n = 202
Demographics	
Male sex, n (%)	163 (80.7)
Age, median (IQR), years	65 (59–69)
ASA classification ≥ III, n (%)	44 (21.8)
Alcohol consumption	
Yes, n (%)	158 (78.2)
Units per week, median (IQR)	21 (14–28)
No, n (%)	44 (21.8)
Alcohol use in the past, n	29
Units per week, median (IQR)	38 (20–70)
Tobacco use	
Current, n (%)	88 (43.6)
Pack years, median (IQR)	40 (30–55)
Former, n (%)	103 (51.0)
Pack years, median (IQR)	40 (20–50)
Never, n (%)	11 (5.4)
HNSCC characteristics	n = 216
HNSCC location 1 , n (%)	
Nasopharynx	1 (0.5)
Hypopharynx	58 (26.9)
Oropharynx	69 (31.9)
Oral cavity	40 (18.5)
Larynx	48 (22.2)
T stage 1 , n (%)	
Tis	18 (8.3)
T1	46 (21.3)
T2	70 (32.4)
T3	46 (21.3)
T4	36 (16.7)
N stage 1 , n (%)	
N0	130 (60.2)
N1	27 (12.5)
N2	4 (1.9)
N2a	5 (2.3)
N2b	31 (14.4)
N2c	13 (6.0)
N3b	6 (2.8)
M0 stage, n (%)	202 (100)
HNSCC treatment, n (%)	
Chemotherapy and/or radiotherapy	131 (64.9)
Surgery	33 (16.3)
Surgery + radiotherapy	17 (8.4)
Surgery + chemoradiotherapy	2 (1.0)
Laser	17 (8.4)
No treatment	2 (1.0)

IQR, interquartile range; ASA, American Society of Anesthesiologists; HNSCC, head and neck squamous cell carcinoma.

1 Calculated for the total number of head and neck squamous cell carcinoma (n = 216), excluding recurrences.

### Endoscopic screening

**Fig. 2:**
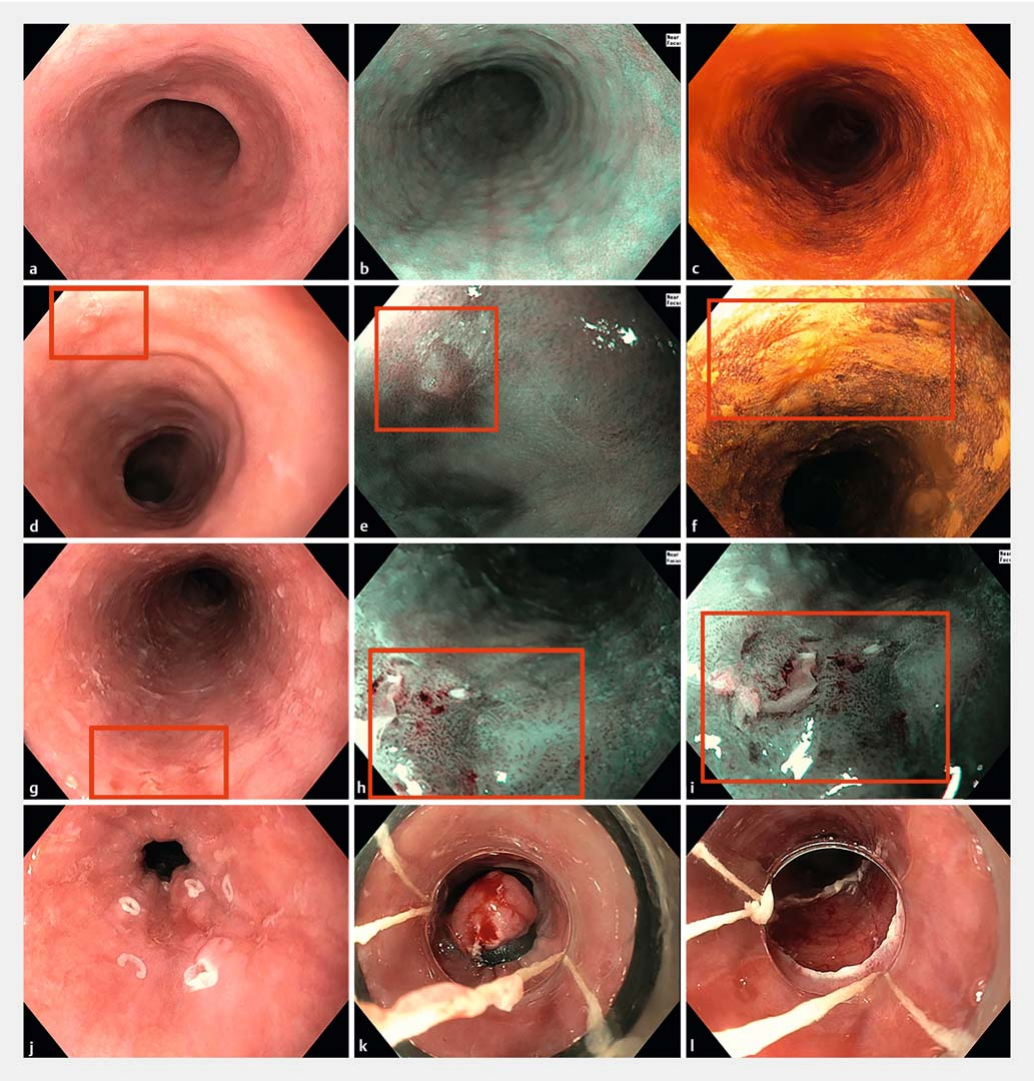
Endoscopic screening of the esophagus. Endoscopic images with white-light endoscopy ( **a** , **d** , **g** , **j–l** ), optical chromoendoscopy ( **b** , **e** , **h** ), and Lugol’s staining ( **c** , **f** ). **a–c** Endoscopic screening of the esophagus without abnormalities. **d–f** Early esophageal squamous cell carcinoma detected during endoscopic screening (patient 5). Endoscopic mucosal resection confirmed a pT1a esophageal squamous cell carcinoma. **g–i** Squamous high grade dysplasia, which could be removed with endoscopic mucosal resection ( **j–l** ) (patient 1).

We performed 250 screening endoscopies of the upper GI tract in 202 patients with HNSCC. First endoscopic screening was performed in all patients and 48 patients underwent follow-up endoscopic screening after 1 year ( **Fig. 2 s** ). In 85 patients, we performed synchronous screening (34.0 % of all screening endoscopies) with a median time between HNSCC diagnosis and screening of 9 days (IQR 6–20 days). Of the synchronously screened patients, 52 (61.2 %) underwent a follow-up endoscopy after 1 year. Indications for the follow-up endoscopy were screening (n = 48) and surveillance after treatment of a synchronous SPT (n = 4). The remaining patients (38.8 %) could not be included in metachronous screening, as these patients were no longer eligible (83.9 %) or did not wish to undergo follow-up screening (16.1 %) ( **Fig. 1 s** ). Subsequently, we performed metachronous screening only in 117 patients. In total, metachronous screening endoscopies (n = 165) were performed 6 months to 1 year (n = 20; 8.0 % of all screening endoscopies), 1–2 years (n = 84; 33.6 %), and 2–5 years (n = 61; 24.4 %) after HNSCC diagnosis. No adverse events occurred as a result of endoscopic screening.

### SPTs detected with endoscopic screening

A total of 11 esophageal SPTs were detected in 10 /202 patients (5.0 %, 95 %CI 2.4 %–8.9 %) during 250 screening endoscopies ( Table 2 , patients 1–10). The SPTs had a median size of 20 mm (IQR 15–30 mm). First endoscopic screening detected 10 SPTs in 9 patients during 202 screening endoscopies (4.5 %). Follow-up endoscopic screening resulted in the detection of 1 SPT during 48 screening endoscopies (2.1 %). During synchronous screening (n = 85), SPTs were detected in six patients (7.1 %). One of the synchronous SPTs was identified during pathology re-assessment of LGD, which was performed by three expert pathologists 1 year after endoscopic resection, revealing HGD (patient 2). During metachronous screening (n = 165), five SPTs were detected in four patients (2.4 %). Metachronous screening performed 1 year after synchronous screening resulted in the detection of one SPT (1/48; 2.1 %), while metachronous screening alone led to the detection of four SPTs in three patients (3/117; 2.6 %). None of the SPTs detected during endoscopic screening (0/11) were detected during the diagnostic work-up (including MRI or PET/CT scan) or routine follow-up for HNSCC prior to endoscopic screening.

**Table 2:** Characteristics of patients with head and neck squamous cell carcinoma and an esophageal second primary tumor, detected during endoscopic screening.

Patient characteristics					HNSCC characteristics		SPT characteristics						
ID	Sex	Age (years)	Alcohol (U/wk)	Smoking (PY)	Sub-location	TN stage	Time to detection, months	Location, cm	Paris classification (diameter, mm)	IPCL + AVA classification	SPT stage	Tx	Outcome (time after SPT diagnosis, months)
1	M	62	21	20	Oropharynx + hypopharynx	T4N2c + T2N2c	0	38	0-IIa (20)	B2 + AVA-middle	HGD	EMR	Endo FU: no recurrence (31)
2	F	67	28	0	Larynx	T2N0	0	24	0-IIb (5)	B1 + no AVA	HGD 1	EMR	Endo FU: no recurrence (12)
3 2	M	67	0	0	Oropharynx	T2N0	0	21–24	0-IIb (30)	B1 + no AVA	HGD	ESD	Endo FU: 2nd esophageal SPT (16); Tx ESD (HGD)
4 2	F	62	9	30	Hypopharynx	T4N2b	0	23–31	0-IIb + 0-IIa (80)	B2 + no AVA	HGD	ESD	Endo FU: 2nd SPT (16); Tx ESD (HGD) 3 rd SPT (31); Tx EMR (pT1a)
5	M	67	14	50	Oral cavity	T2N0	18	30	0-IIa (15)	B2 + no AVA	T1a	EMR	Endo FU has been planned
6	M	74	20	10	Oral cavity	T2N0	15	21	0-IIa (10)	B2 + no AVA	T1a	EMR	No FU (patient wish)
7	M	67	0	13	Oropharynx + hypopharynx	T2N2c + T2N2c	0	20	0-IIa (20)	B2 + no AVA	T1a	ESD + CRT	No FU. Patient died (19) due to palliative HNSCC
8	F	77	3	43	Oropharynx	T4aN2	32	15–17	0-IIa + 0-IIc (15)	B2 + AVA-large	T1b	ESD	No FU, because no Tx options in case of recurrence
9	M	48	42	31	Hypopharynx	T2N2c	0	20	0-Is + 0-II (20)	-	T2	CRT	Recurrence ESCC (9): Tx surgery Patient died (18) due to surgical complications
10	M	69	48	100	Hypopharynx	T1N3b	27 27	27–28 18–22	0-IIa + 0-IIc (15) Stricturing tumor (40)	-	T2 T4	RT	Patient died (6) due to ESCC

HNSCC, head and neck squamous cell carcinoma; SPT, second primary tumor; U/wk, units/week; PY, pack years; TN, tumor, node; IPCL, intrapapillary capillary loops according to the Japanese Esophageal Society classification; AVA, avascular areas; Tx, treatment; HGD, high grade dysplasia; EMR, endoscopic mucosal resection; endo, endoscopic; FU, follow-up; ESD, endoscopic submucosal dissection; CRT, chemoradiotherapy; ESCC, esophageal squamous cell carcinoma; RT, radiotherapy.

1 In the previously published synchronous screening study [3], the grade of dysplasia was reported as low grade dysplasia. Pathological re-assessment was performed by three expert pathologists after 1 year and revealed HGD.

2 Only the characteristics of the first SPT in the upper gastrointestinal tract are shown for this patient.

### Increased detection of early stage SPTs with endoscopic screening

Of the 10 patients with an SPT, 90.0 % were diagnosed with an early stage SPT ( Table 2 , patients 1–9). The SPTs in patients 1–8 were treated with endoscopic resection (EMR n = 4, ESD n = 4) with curative intent ( Table 2 , Fig. 2 ). Histopathological assessment of the resection specimen showed HGD (n = 4), and pT1a (n = 3), and pT1b (n = 1) cancer. In two patients, the radiotherapy field for HNSCC was extended to include a synchronous esophageal SPT because of the presence of lymphovascular invasion in the endoscopic resection specimen (patient 5) and for a T2 SPT (patient 9). One patient without clinical signs of dysphagia or odynophagia was diagnosed with both a T4 and T2 SPT during endoscopic screening (patient 10). Besides the detection of SPTs, LGD was detected in two patients (1.0 %) and treated with EMR in one patient. The second patient died due to HNSCC before endoscopic resection was performed.

### Other relevant GI findings detected with endoscopic screening

During endoscopic screening, one patient was diagnosed with an esophageal adenocarcinoma and one patient was diagnosed with gastric cancer. Both patients could be treated curatively with endoscopic resection (EMR n = 1, ESD n = 1), and histopathological assessment revealed T1a cancer (n = 2). Both patients received endoscopic follow-up without recurrence. The patient diagnosed with esophageal adenocarcinoma was also treated with radiofrequency ablation. Other findings included the presence of gastroesophageal reflux disease (13.4 %; grade A in 5.0 %, grade B in 5.9 %, grade C in 1.0 %, and grade D 0.5 %), Barrett’s esophagus (10.4 %), and gastric intestinal metaplasia or confirmed *Helicobacter pylori* infection (5.4 %).

### Endoscopic detection techniques

**Fig. 3:**
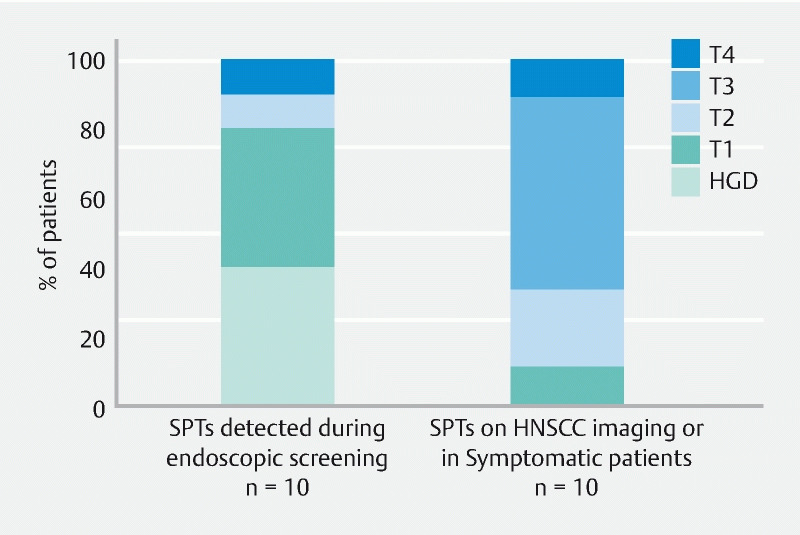
Tumor stage of second primary tumors (SPTs) of eligible patients with head and neck squamous cell carcinoma. For one patient with two SPTs detected during endoscopic screening (patient 10), only the most advanced SPT was shown. The tumors stage of one patient (patient 20) was unknown. HGD, high grade dysplasia ; HNSCC, head and neck squamous cell carcinoma

Confirmed SPTs in the esophagus were detected with WLE (9/11), NBI (10/11), and Lugol’s staining (6/7) ( Table 3 ). No Lugol’s staining was used in the assessment of four SPTs, as it was deemed not to have additional diagnostic value in the SPT diagnosis. 10/11 SPTs were detected with WLE combined with NBI. The additional value of Lugol’s staining after WLE and NBI in expert hands was the detection of HGD in one patient and LGD in one patient. The positive predictive value was the highest for NBI (57.9 %) and lowest for Lugol’s staining (15.7 %). The false-positive detection rate of Lugol’s staining was 84.3 %. **Fig. 3 s** depicts different Lugol voiding lesions detected during endoscopic screening, with corresponding grades of dysplasia confirmed during pathological assessment.

**Table 3:** Detection of second primary tumors and low grade dysplasia in the upper gastrointestinal tract with different endoscopic screening techniques.

	WLE	NBI	Lugol’s staining
Total screening endoscopies n	250	249 1	238 2
Total suspected lesions n (%)	18 lesions during 15 (6.0 %) endoscopies	19 lesions during 16 (6.4 %) endoscopies	52 lesions during 38 (16.0 %) endoscopies
Pathology of suspected lesions, n/N 3			
ESCC	7/7	7/7	2/3 ^2^
HGD	2 /4	3 /4	4 /4
LGD	1 /2	1 /2	2 /2
No dysplasia	8	8	43
No pathology	0	0	1
Positive predictive value, n/N (%)			
For the detection of an SPT	9/18 (50.0)	10/19 (52.6)	6/51 (11.8) 4
For the detection of an SPT/LGD	10/18 (55.6)	11/19 (57.9)	8/51 (15.7) 4
False positives	8/18 (44.4)	8/19 (42.1)	43/51 (84.3) 4

WLE, white-light endoscopy; NBI, narrow-band imaging; ESCC, esophageal squamous cell carcinoma; HGD, high grade dysplasia; LGD, low grade dysplasia; SPT, second primary tumor.

1 No NBI was used during one endoscopy owing to patient discomfort.

2 No Lugol’s staining was used during 12 endoscopies because it had no additional diagnostic value for the assessment of SPTs, patient discomfort, or allergy.

3 Number of SPTs detected with endoscopic screening technique/total number of SPTs detected in the included patients.

4 Calculated for the total number of lesions with pathological confirmation (n = 51). Patients with nonsquamous lesions, including esophageal adenocarcinoma or gastric cancer, are not shown (n = 2).

### SPTs detected on HNSCC imaging and in symptomatic patients

Among patients eligible for metachronous screening only (n = 389, **Fig. 1 s** ), 10 patients with HNSCC had already been diagnosed with an esophageal SPT before these patients could be approached for endoscopic screening ( **Table 2 s** , patients 11–20). These SPTs were detected during the HNSCC diagnostic work-up (n = 6) and follow-up (n = 1), and in patients with symptoms of dysphagia and odynophagia (n = 3). Unlike the SPTs in screened patients with HNSCC, SPTs among those not screened were detected more often at advanced stages (50.0 %) ( Fig. 3 ) and no SPTs could be treated with endoscopic resection. Esophageal SPT-related deaths occurred in 6/10 patients, all within 12 months after SPT diagnosis.

## Discussion

Endoscopic screening in patients with HNSCC holds the potential to detect SPTs in the esophagus at early stages. Currently, no routine screening for SPTs in patients with HNSCC has been implemented in most Western countries, and the yield and benefit of endoscopic screening are yet to be determined 13 21 . We conducted a prospective endoscopic screening study and detected SPTs in 5 % of 202 patients with HNSCC in the Netherlands. Most SPTs were detected at an early stage and could be treated curatively with endoscopic resection.

Our SPT prevalence of 5 % is in line with previous endoscopic screening studies originating from European countries, reporting a prevalence ranging from 3 % to 10 % SPTs in patients with HNSCC 15 16 17 . We also reported on other GI tract cancers detected during endoscopic screening. Although risk profiles of different types of cancer in the upper GI tract differ strongly, we believe that these cancers should also be reported in Western screening studies for SPTs. The incidence of esophageal adenocarcinomas is rising in Western countries 8 , and early detection of upper GI tract cancers potentially has substantial positive consequences with regard to prognosis and survival of patients with HNSCC.

Screening in patients with HNSCC should focus on the detection of SPTs at early stages, as timely detection of SPTs may improve the survival rates of these patients. Previous literature assessing the use of PET/CT as the screening modality for detection of SPTs reported a limited sensitivity of up to 38 %, particularly for the detection of early stage esophageal cancers 13 23 24 . This is in line with our study, as none of the SPTs detected on routine cross-sectional imaging for HNSCC were detected at early stages or could be treated with endoscopic resection. In contrast, 80 % of the patients with SPTs detected during endoscopic screening could be treated with endoscopic resection.

The frequency and timing are key aspects of endoscopic screening in patients with HNSCC. Based on current data, one-time endoscopic screening may be preferable over repeat endoscopic screening, as follow-up endoscopic screening in synchronously screened patients had a relatively limited SPT yield of 2 %. The timing of one-time endoscopic screening should be further investigated, as synchronous endoscopic screening performed as part of the HNSCC diagnostic work-up has the potential to discover asymptomatic SPTs at the earliest stage possible. In the current study, however, 22 % of synchronously screened patients developed metastatic HNSCC within 1 year after diagnosis and therefore did not benefit from synchronous screening. An advantage of metachronous screening is that a smaller selection of HNSCC patients with a favorable prognosis from HNSCC remain. Screening a smaller selection of HNSCC survivors is likely to be more cost effective than screening the entire HNSCC population and these patients probably have more benefit from early detection of SPTs. Therefore, a key aspect of the timing of screening is HNSCC-related survival rates, which depend on HNSCC staging and subsite. The 2-year survival rates vary between 62 % for hypopharyngeal and oropharyngeal cancer, to 81 % for laryngeal cancer 25 . Based on previous literature and current data, we hypothesize that the optimal timing of screening might potentially be 1–2 years after HNSCC diagnosis, whereas potentially synchronous SPTs are still discovered at curable stages.

In the current study, systematic endoscopic screening was performed with WLE, NBI, and Lugol’s staining. In expert hands using high definition endoscopes, Lugol’s staining often resulted in additional biopsies and endoscopic resections, while the detection of additional SPTs was limited. These results are in line with the 2022 update of the European Society of Gastrointestinal Endoscopy, which recommends the use of high definition endoscopy with WLE and NBI to screen for esophageal neoplasia 26 .

Although this was a large endoscopic screening study in patients with HNSCC in a Western country, some limitations need to be addressed. This was a single-center study including a selection of patients with HNSCC with presumed highest risk of SPTs based on previous Asian studies. This may limit the generalizability to all patients with HNSCC in daily clinical practice. In the Netherlands, care for patients with HNSCC is centralized in 14 expert centers with uniform staging and treatment. We therefore expect that our results also apply to patients in other Western expert HNSCC centers with experienced endoscopists. However, awareness and perspectives regarding endoscopic screening for SPTs may differ between specialists 27 .

The timing of endoscopic screening differed between included patients. Further studies should investigate individual risk–benefit profiles of all patients with HNSCC in Western countries. The ideal setting would be the combination of a nationwide endoscopic screening and the development of a risk prediction model, both including all patients treated curatively for HNSCC. Based on current guidelines, endoscopic screening should be performed with WLE and NBI. Lugol’s staining may potentially be used based on endoscopists’ preference.

In conclusion, endoscopic screening resulted in the detection of an esophageal SPT in 5 % of patients with HNSCC. Most SPTs were detected at an early stage and could be treated with curative intent. Therefore, endoscopic screening for SPTs should be considered in selected patients with HNSCC. This selection should include patients with highest risk for SPTs (e. g. alcohol and tobacco consumption, hypopharyngeal and human papillomavirus-negative oropharyngeal carcinomas) with an acceptable life expectancy according to HNSCC prognosis and comorbidities. Metachronous one-time screening after curative treatment and adequate follow-up time seems preferable for patients with HNSCC in Western countries. Based on our data, combined with selection of patients with favorable survival prospects, we suggest a timing of between 12 and 24 months after HNSCC diagnosis.

## Tables 1 s, 2s, Fig. 1 s–3 s

Supplementary material Zusatzmaterial
