# Supplementary material for: Endoscopic screening of the upper gastrointestinal tract for second primary tumors in patients with head and neck cancer in a Western country
Source: Endoscopy. 2023 Jul 25;55(11):981–90. doi: 10.1055/a-2111-5935 (PMC10602659; doi:10.1055/a-2111-5935)
Supplement: Supplementary file 1 — Supplementary material [file 22888supmat_10-1055-a-2111-5935.pdf]

Supplementary material

Endoscopic screening of the upper gastrointestinal tract for second primary tumors in patients with head and neck cancer in a Western country

Laurelle van Tilburg, Steffi E.M. van de Ven, Pieter Jan F. de Jonge, Wilmar de Graaf, Manon C.W. Spaander, Suzan Nikkessen, Jose A. Hardillo, Aniel Sewnaik, Dominiek A. Monserez, Hetty Mast, Stijn Keereweer, Marco J. Bruno, Robert J. Baatenburg de Jong, Arjun D. Koch

Supplementary material

Table 1s. Baseline and HNSCC characteristics of the included patients with (n=10) and without (n=192) an esophageal SPT, detected during endoscopic screening.

Patient characteristics	Patients without SPT, n=192	Patients with SPT, n=10
**Demographics**		
Male sex	156 (81.3%)	7 (70.0%)
Age, years	65 [58-69]	67 (62-68)
ASA classification ≥III	42 (21.9%)	2 (20.0%)
**Alcohol consumption**		
Yes	150 (78.1%)	8 (80.0%)
Units per week	21 (14-35)	25 [10-39]
No	42 (21.9%)	2 (20.0%)
Alcohol use in the past	27	2
Units per week	40 [20-70]	84 [84-84]
**Tobacco use**		
Current	80 (41.7%)	8 (80.0%)
Pack years	40 [30-55]	31 [23-50]
Former	102 (53.1%)	1 (10.0%)
Pack years	40 [20-50]	50 [50-50]
Never	10 (5.2%)	1 (10.0%)
**HNSCC characteristics**	n=204	n=12
**HNSCC location** [1]		
Nasopharynx	1 (0.5%)	0
Hypopharynx	53 (26.0%)	5 (41.7%)
Oropharynx	65 (31.9%)	4 (33.3%)
Oral cavity	38 (18.6%)	2 (16.7%)
Larynx	47 (23.0%)	1 (8.3%)
**T stage** [1]		
Tis	18 (8.8%)	0
T1	45 (22.1%)	1 (8.3%)
T2	63 (30.9%)	7 (58.3%)
T3	45 (22.1%)	1 (8.3%)
T4	33 (16.2%)	3 (25.0%)
**N stage** [1]		
N0	126 (61.8%)	4 (33.3%)
N1	27 (13.2%)	0
N2/N2a/N2b/N2c	3 (1.5%) / 5 (2.5%) / 28 (13.7%) / 9 (4.4%)	1 (8.3%) / 0 / 3 (25.0%) / 4 (33.3%)
N3b	6 (2.9%)	0
**M stage**		
M0	192 (100%)	10 (100%)
**HNSCC treatment**		
Chemotherapy and/or radiotherapy	124 (64.5%)	7 (70.0%)
Surgery	31 (16.1%)	2 (20.0%)
Surgery + radiotherapy	16 (8.3%)	1 (10.0%)
Surgery + chemoradiotherapy	2 (1.0%)	0
Laser	17 (8.9%)	0
No treatment	2 (1.0%)	0

Data presented as n with percentage or median [p25-p75]. [1] Calculated for the total number of head and neck squamous cell carcinoma with SPTs (n=12) and without SPTs (n=204), excluding recurrences. HNSCC, head and neck squamous cell carcinoma; SPT, second primary tumor.

Supplementary material

Table 2s Characteristics of patients with HNSCC and an esophageal SPT, diagnosed on HNSCC imaging or in symptomatic patients (n=10)

Patient			HNSCC characteristics		SPT characteristics						
ID	Sex	Age	Sub-location	TN stage	Symptoms	Indication	Primary detection method	Time to detection (months)	Tumor stage	Treatment	Outcome (follow-up period in months)
11	M	67	Larynx	T3N2c	Dysphagia	HNSCC diagnostic work-up	PET/CT	2	T1	CRT + surgery	Patient died (15), due to ESCC or HNSCC
12	M	65	Larynx	T3N2c	None	HNSCC diagnostic work-up	CT	0	T2	CRT	No recurrence (24)
13	M	55	Oropharynx	T4aN2c	Dysphagia + odynophagia	HNSCC diagnostic work-up	PET/CT	1	T2	CRT	No recurrence (53)
14	F	78	Oropharynx	T1N2c	None	HNSCC diagnostic work-up	CT	1	T3	RT	Patient died (9), due to ESCC
15	M	62	Oropharynx	T2N3	None	HNSCC diagnostic work-up	CT	0	T3	No treatment	Patient died (7), due to ESCC
16	F	70	Oropharynx + oral cavity	T2N0 + T1bN0	None	HNSCC diagnostic work-up	PET/CT	37	T3	RT	Patient died (5), due to ESCC
17	M	54	Hypopharynx	T3N2b	Dysphagia + odynophagia	Symptoms	CT	35	T3	RT	Patient died (3), due to ESCC
18	M	65	Oropharynx	T3N2a	Dysphagia	Symptoms	CT	11	T3	CRT	Patient died (10), due to ESCC
19	M	62	Hypopharynx	T2N2c	Odynophagia	HNSCC recurrence	PET/CT	21	T4a	RT	Patient died (5), due to ESCC
20	F	67	Hypopharynx	T3N0	Dysphagia + odynophagia	Symptoms	Endoscopy	19	Tx	CRT	No recurrence (30)

CT, computed tomography; CRT, chemo- and radiotherapy; ESCC, esophageal squamous cell carcinoma; HNSCC, head and neck squamous cell carcinoma; PET/CT, Positron Emission Tomography/Computed Tomography; SPT, second primary tumor; RT, radiotherapy.

Supplementary material

Fig. 1s Flowchart of patient inclusion in the synchronous and metachronous endoscopic screening.

HNSCC, head and neck squamous cell carcinoma; GI, gastrointestinal; HPV, human papillomavirus; SPT, second primary tumor. [1] Also included in the synchronous endoscopic screening study, published by van de Ven et al. (2021). The time between HNSCC diagnosis and endoscopic screening was divided in synchronous (without 6 months) and metachronous (after more than 6 months).

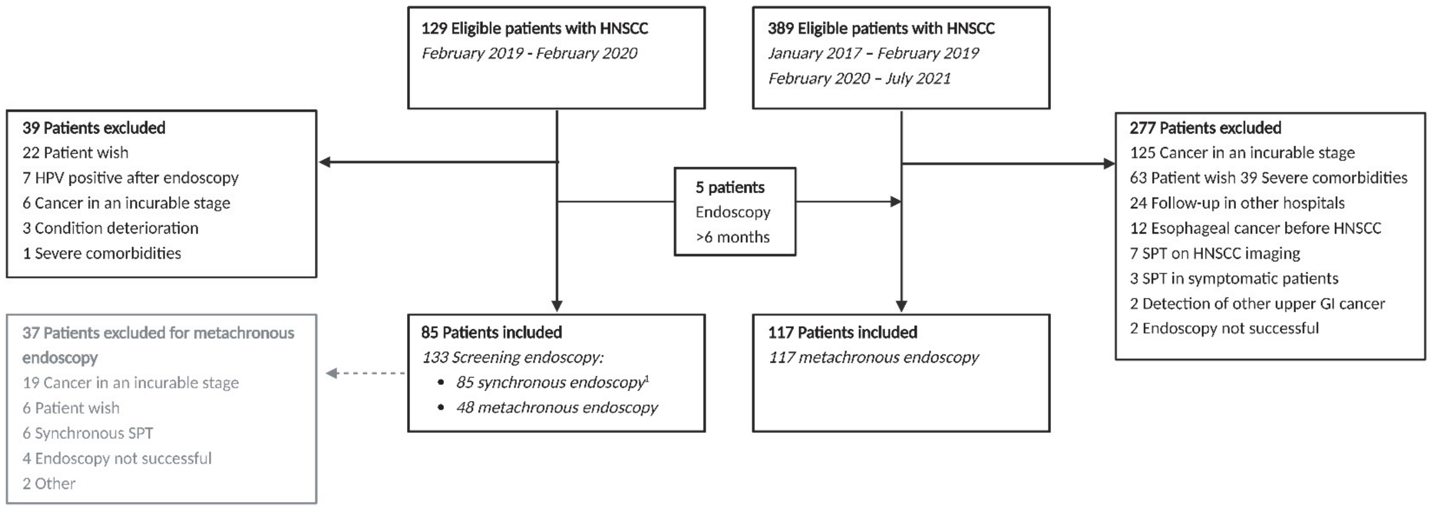

Supplementary material

Fig. 2s Timing of endoscopic screening of the upper gastrointestinal tract in patients with head and neck cancer.

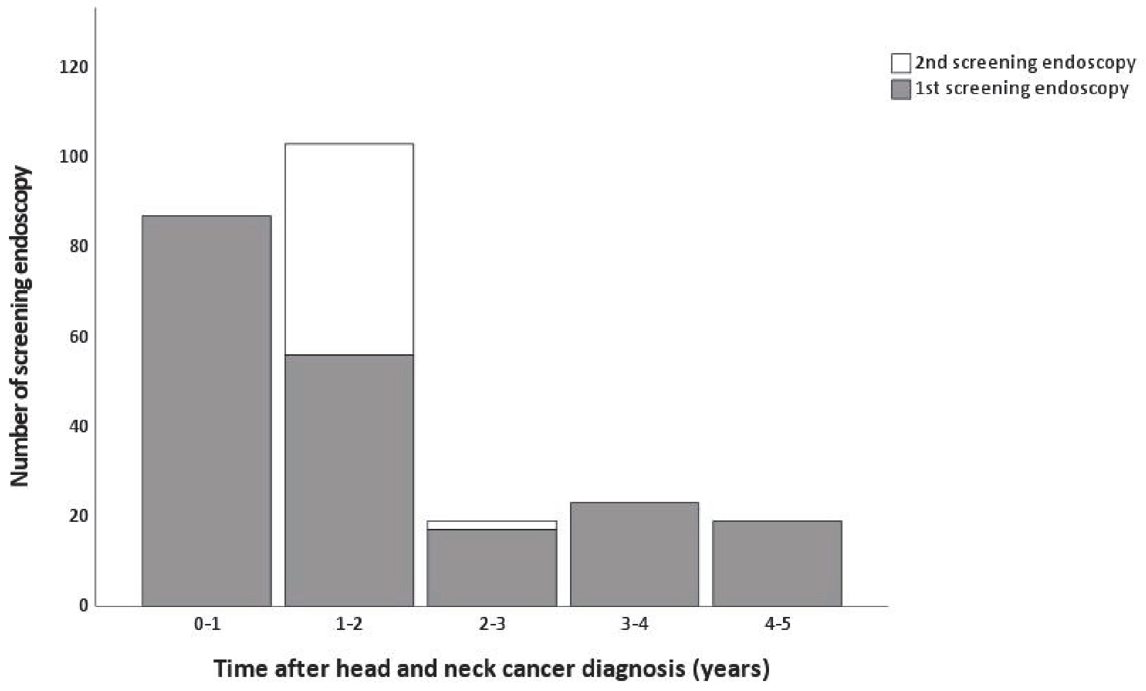

Thieme

Supplementary material

Fig. 3s Endoscopic images of Lugol voiding lesions, detected during endoscopic screening of the esophagus.

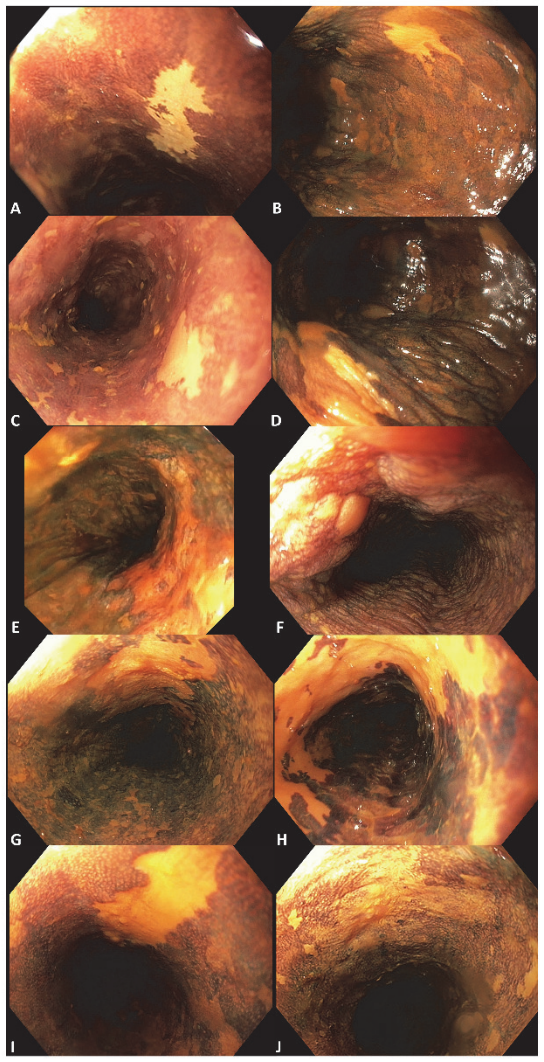

Endoscopic images of Lugol voiding lesions in the esophagus, detected during endoscopic screening in patients with HNSCC. Most lesions were removed with endoscopic resection (all lesions except E). The pathology assessment revealed no dysplasia for the lesions showed in images A-D, low-grade dysplasia for lesions E and F, high-grade dysplasia for lesions G and H and a T1a esophageal squamous cell carcinoma for I and J.
